# Exodus: sequencing-based pipeline for quantification of pooled variants

**DOI:** 10.1093/bioinformatics/btac319

**Published:** 2022-05-12

**Authors:** Ilya Vainberg-Slutskin, Noga Kowalsman, Yael Silberberg, Tal Cohen, Jenia Gold, Edith Kario, Iddo Weiner, Inbar Gahali-Sass, Sharon Kredo-Russo, Naomi B Zak, Merav Bassan

**Affiliations:** BiomX Ltd., Ness Ziona 7414002, Israel; BiomX Ltd., Ness Ziona 7414002, Israel; BiomX Ltd., Ness Ziona 7414002, Israel; BiomX Ltd., Ness Ziona 7414002, Israel; BiomX Ltd., Ness Ziona 7414002, Israel; BiomX Ltd., Ness Ziona 7414002, Israel; BiomX Ltd., Ness Ziona 7414002, Israel; BiomX Ltd., Ness Ziona 7414002, Israel; BiomX Ltd., Ness Ziona 7414002, Israel; BiomX Ltd., Ness Ziona 7414002, Israel; BiomX Ltd., Ness Ziona 7414002, Israel

## Abstract

**Summary:**

Next-Generation Sequencing is widely used as a tool for identifying and quantifying microorganisms pooled together in either natural or designed samples. However, a prominent obstacle is achieving correct quantification when the pooled microbes are genetically related. In such cases, the outcome mostly depends on the method used for assigning reads to the individual targets. To address this challenge, we have developed Exodus—a reference-based Python algorithm for quantification of genomes, including those that are highly similar, when they are sequenced together in a single mix. To test Exodus’ performance, we generated both empirical and *in silico* next-generation sequencing data of mixed genomes. When applying Exodus to these data, we observed median error rates varying between 0% and 0.21% as a function of the complexity of the mix. Importantly, no false negatives were recorded, demonstrating that Exodus’ likelihood of missing an existing genome is very low, even if the genome’s relative abundance is low and similar genomes are present in the same mix. Taken together, these data position Exodus as a reliable tool for identifying and quantifying genomes in mixed samples. Exodus is open source and free to use at: https://github.com/ilyavs/exodus.

**Availability and implementation:**

Exodus is implemented in Python within a Snakemake framework. It is available on GitHub alongside a docker containing the required dependencies: https://github.com/ilyavs/exodus. The data underlying this article will be shared on reasonable request to the corresponding author.

**Supplementary information:**

Supplementary data are available at *Bioinformatics* online.

## 1 Introduction

Next-Generation Sequencing (NGS)-based protocols have become prevalent across many domains of life sciences (Levin *et al.*, 2021; Schweiger *et al.*, 2011). A common task addressed by NGS is determination of the relative abundances of multiple microorganisms mixed together in the same sample (David *et al.*, 2014; Martin *et al.*, 2020). This is often achieved by competitively mapping the reads obtained from the NGS-based experiment to a set of reference genomes and transforming the coverage vectors on each reference to relative abundance values. While such NGS-based protocols offer many advantages, such as high throughput and generalism, a major shortcoming is inability to differentiate between genetically similar components. In cases of samples with similar components, the vast majority of reads will be uninformative, as they could equally be mapped to more than one reference. Naively using the remaining reads will result in inaccurate results.

In the field of microbiome research, where samples are typically composed of complex bacterial communities, taxonomic assignment algorithms such as Kraken (Wood and Salzberg, 2014), MetaPhlAn (Segata *et al.*, 2012) and CoreProbe (Ai *et al.*, 2018) are commonly used to match the NGS data to large public databases at either genus or species level. Notably, there are tools that were built to achieve strain resolution from metagenomic data, such as TAEC (Sohn *et al.*, 2014) which uses genomic similarity to correct the initial taxonomic assignment, and TAMER (Jiang *et al.*, 2012) which uses a mixture model to assign reads to detected genomes.

However, these tools are incongruent with non-microbiome use cases, such as the development of biotherapeutic products. In such use cases the reference genomes are not necessarily bacterial [for example phage (Górski *et al.*, 2020) or yeast (Mimee and Nagler, 2021)], not necessarily registered in public databases, their degree of similarity to other potentially present genomes can be high (sub-strain level), and still the error rate must be kept low enough to comply with regulatory bodies. These problems are often circumvented by devising *ad* *hoc* experimental setups, such as targeted-NGS (Zhou *et al.*, 2011) or custom DNA-chips (Quackenbush, 2001), however these solutions require specific calibrations and thus undermine the generality of the NGS-based approach. To overcome this tradeoff, we developed Exodus: an algorithm for accurate quantification of highly similar custom references based solely on whole genome NGS.

## 2 Algorithm description

**Fig. 1.**
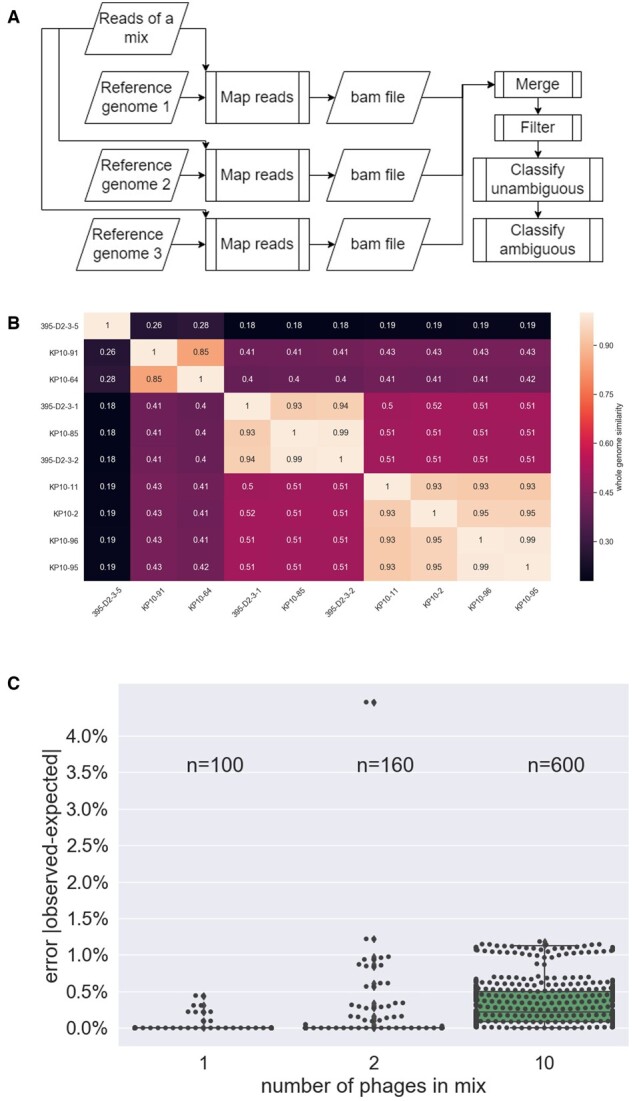
Description and performance of the Exodus Algorithm. (**A**) Schematic flow chart of the Exodus algorithm. (**B**) Distance matrix based on global alignment (Rice *et al.*, 2000) between all genomes used to benchmark Exodus. (**C**) Distribution of errors (expressed as the absolute value of the margin between expected and observed) in Exodus’ performance, as a function of the number of genomes that were present in the sample

The Exodus algorithm is a multistep process (Fig. 1A) implemented in Python using a Snakemake workflow (Köster and Rahmann, 2012); thus it runs in parallel on the available resources of the machine at hand with no user intervention required.

### 2.1 Input

Exodus receives a configuration file containing (i) paths to standard paired end short read sequencing fastq files and (ii) the paths to the reference genomes of the organisms to be quantified.

### 2.2 Read filtering and mapping

Manually pre-processing the reads is recommended but depends on the use case and the experimental setup (see documentation on GitHub for more info). Exodus maps the reads to each of the given reference genomes individually, using BWA mem (Li and Durbin, 2009) with default parameters. The resulting binary alignment map (BAM) files (Li *et al.*, 2009) are merged and filtered to keep only properly paired reads where both reads of a pair had the same reference with the highest alignment score.

### 2.3 Assignment of unique reads

The merged BAM file is traversed in search of reads that perfectly align to exactly one reference. These reads are assigned to their matching references, and the temporary Relative Abundance (RA) of each reference is computed:

$$\begin{array}{c} C_{\mathrm{ref}}=\frac{mbp_{\mathrm{ref}}}{g_{\mathrm{ref}}}\\ RA_{\mathrm{ref}}=\frac{C_{\mathrm{ref}}}{\sum_{i=1}^{n}C_{i}} \end{array},$$

where *C* is the coverage of a quantified reference, *mbp* is the number of bases mapped to a quantified reference, *g* is the genome length of a quantified reference, *n* is the total number of references in the given reference set, and *i* is an index representing a reference.

### 2.4 Assignment of tied reads

In the next step reads that perfectly align to more than one reference are assigned to one of the tied references in a ratio matching the relative abundance between the references, as found in the previous step. In this step, Exodus handles special cases where one of the tied references has exactly zero perfect unique reads from the earlier step; these cases are mapped out and the reads are all assigned to the reference that did have unique reads. Subsequently relative abundance is re-computed before moving on to the resolving other ties. After assigning all perfectly matched reads (both unique and ties), Exodus computes that final relative abundances (using the same equations described above), and outputs them alongside a report with crude data from the run.

## 3 Algorithm performance

### 3.1 Accuracy

Bacteriophage were used in this study for algorithm validation purposes due to their quick growth, compact genomes and varying levels of genomic similarity. Thus, to test Exodus we chose 10 double-stranded DNA bacteriophages (genome sizes are given in Supplementary Table S1) presenting an array of genomic distances (Fig. 1B). Empirical data was generated by mixing phage pairs in known ratios. Phage DNA was extracted by Phage DNA Isolation Kit (cat 46800). Library preparation was performed using Illumina’s Tagment DNA Enzyme & Buffer (cat 20034197) (Baym *et al.*, 2015), and libraries were sequenced on an Illumina Miseq v2 PE250 platform, with roughly 100 000 reads/sample. In addition, *in* *silico* data was generated by simulating reads for the same phage genomes mentioned above using ART (Huang *et al.*, 2012) with similar depth and error profiles as obtained from the empirical sequencing data. These simulations allowed us to test a wide array of phage combinations, including complex cases of multiple phage and small ratios—which are challenging to produce empirically, but can occur naturally in real samples (Supplementary Table S2 shows all the empirical and simulated combinations tested).

Each sample was analyzed by Exodus where the reference set was always comprised the same 10 phage genomes. To determine the quantification error of each genome in a sample, we simply computed the absolute value of the margin between the expected and observed relative abundance values. The median error over 860 observations was 0.12%. While the number of phages in the mix was positively correlated with the error rate (higher error rates for samples with more phage), the effect size was still small in the mixes comprising 10 phages (Fig. 1C).

False negative rate was defined as cases where Exodus did not assign a positive relative abundance value to samples that were present in the mix. Out of 632 cases where the true relative abundance of a genome was positive, no false negative cases were observed. False positive rate was defined as the relative abundance Exodus assigned for reference genomes that were absent from the sample. Exodus’ maximum and median false positive rates were 0.4% and 0%, respectively. Out of 228 cases where a genome was not present in the sample, Exodus correctly assigned its relative abundance to zero in 211 cases (Supplementary Table S3).

### 3.2 Resource usage

Resource utilization was tested using our empirical data (Supplementary Table S2) by varying the number of NGS samples included in a single analysis run from one to 26 with the 10 relevant phage genomes in the reference set. The benchmarking was run on AWS EC2 c5d.12xlarge instances. In such setup, Exodus’ peak RAM usage was ∼69 mb and the runtime was a linear function of the number of samples varying between 30 to 298 seconds (Supplementary Table S4).

## 4 Conclusion

In this work, we describe the development of Exodus—a reference-based tool for quantification of genomes sequenced together in a single mix. To test Exodus’ performance, we generated both empirical NGS data and *in* *silico* simulations of complex cases where multiple similar genomes in small relative abundances were mixed and sequenced together. We observed that Exodus’ median error rate varied between 0% and 0.21% as a function of the complexity of the mix. Subsequently we observed that false identification of a genome was very rare (7% of cases) and was limited to extremely low relative abundance values, thus users could consider applying a minimum relative abundance threshold as a noise filter. Notably, being a reference-based tool, Exodus is not built to analyze unknown cultures. However, based on this low false identification rate, in cases of uncertainty we recommend including potentially relevant genomes to the reference set, as the noise introduced by excessive references is minimal. Importantly, no false negatives were recorded, demonstrating that Exodus’ likelihood of missing an existing genome is very low, even if the genome’s relative abundance is low and similar genomes are sequenced with it in the same mix. Taken together, these data position Exodus as a reliable tool for identifying and quantifying reference genomes in mixed samples. Immediate use cases for Exodus include detection and quantification of known virus variants in environmental samples (Martínez-Puchol *et al.*, 2021) or verification of the proper composition of a microbiological product, such as probiotics (Williams, 2010) or phage cocktails designed for therapeutic use (Górski *et al.*, 2020).

*Financial Support*: none declared.

*Conflict of Interest*: All authors are current or past employees of BiomX Ltd.

## Supplementary Material

btac319_Supplementary_Data
